# Supplementary material for: The Effects of Iron Deficiency on the Gut Microbiota in Women of Childbearing Age
Source: Nutrients. 2023 Jan 30;15(3):691. doi: 10.3390/nu15030691 (PMC9919165; doi:10.3390/nu15030691)
Supplement: Supplementary file 1 [file nutrients-15-00691-s001.zip › nutrients-2178820-supplementary.pdf]

Supplementary data

Table S1. Distributions of bacterial community at different taxonomic levels.

Rank	Taxa	Normal (%)	PRE (%)	Post (%)
Phylum	*Proteobacteria*	0.0	6.2	2.6
	Actinobacteria	8.7	8.6	9.8
	Bacteroidetes	33.8	35.7	34.1
	Firmicutes	52.6	45.1	53.0
	Verrucomicrobia	0.0	3.2	0.0
	ETC (<1%)	1.0	1.2	0.5
Class	*Alphaproteobacteria*	1.2	2.6	0.0
	Bacteroidia	33.7	35.7	34.1
	Bacilli	1.2	1.1	1.6
	Actinobacteria_c	5.2	6.5	8.3
	Coriobacteriia [*, c]	3.5	2.0	1.5
	Negativicutes [*, a]	4.5	8.8	8.4
	Betaproteobacteria [*, b]	1.5	2.2	1.5
	Erysipelotrichi [*, b]	1.2	1.4	0.0
	Clostridia [*, a]	45.6	33.8	42.2
	Verrucomicrobiae	0.0	3.0	0.0
	ETC (<1%)	2.3	2.8	2.4
Order	*Bacteroidales*	33.7	35.7	34.1
	Burkholderiales	1.5	2.2	1.5
	Clostridiales [*, a]	45.6	33.8	42.2
	Veillonellales	1.3	5.0	5.4
	Rhodospirillales	1.2	2.6	0.0
	Coriobacteriales [*, c]	3.5	2.0	1.5
	Acidaminococcales	1.0	1.7	2.6
	Erysipelotrichales [*, b]	1.2	1.4	0.0
	Selenomonadales	2.2	2.1	0.0
	Bifidobacteriales	5.1	6.2	8.2
	Lactobacillales	1.2	1.1	1.6
	Verrucomicrobiales	0.0	3.0	0.0
	ETC (<1%)	2.6	3.2	3.0
Family	*Christensenellaceae*	1.9	1.1	0.0
	Bifidobacteriaceae	5.1	6.2	8.2
	Rikenellaceae	4.2	3.3	4.0
	Rhodospirillaceae	1.1	2.5	0.0
	Bacteroidaceae	17.8	20.9	18.4
	Prevotellaceae	8.6	8.4	8.3
	Veillonellaceae	1.3	5.0	5.4

	Selenomonadaceae	2.2	2.1	0.0
	Acidaminococcaceae	1.0	1.7	2.6
	Sutterellaceae	1.4	2.2	1.4
	Porphyromonadaceae	1.8	1.8	2.4
	Coriobacteriaceae [*, c]	3.5	2.0	1.5
	Lachnospiraceae	12.0	12.6	11.8
	Erysipelotrichaceae [*, b]	1.2	1.4	0.0
	Ruminococcaceae [**, a]	31.2	19.6	29.1
	Akkermansiaceae	0.0	3.0	0.0
	ETC (<1%)	5.5	6.1	7.0
Genus	*Ruminococcus_g2*	1.8	0.0	1.3
	Subdoligranulum	1.6	0.0	1.5
	Oscillibacter	4.3	3.8	4.1
	Collinsella [*, c]	2.6	1.2	0.0
	Sutterella	1.2	1.9	1.2
	Prevotella	7.7	7.5	7.8
	Lachnospira	1.6	1.5	1.5
	Blautia	1.3	1.6	1.3
	PAC001207_g	1.1	0.0	0.0
	Clostridium_g24	1.0	1.5	0.0
	Sporobacter	1.2	0.0	1.1
	Bifidobacterium	5.0	6.2	8.1
	Eubacterium_g23	2.4	1.3	1.7
	Megamonas	2.2	2.0	0.0
	Roseburia	3.0	1.8	2.4
	Alistipes	4.2	3.3	4.0
	Bacteroides	17.8	20.9	18.3
	Parabacteroides	1.8	1.8	2.4
	Pseudoflavonifractor	1.4	1.7	2.0
	Ruminococcus	1.0	0.0	0.0
	LARJ_g	1.1	2.5	0.0
	Faecalibacterium [**, a]	15.3	7.5	14.9
	Veillonella [*, c]	0.4	2.0	3.9
	ETC (<1%)	19.0	30.0	22.5

Relative abundances less than 1 % were expressed as ETC. Wilcoxon rank-sum test was used to analyze the significance between the groups (*, $p < 0.05$; **, $p < 0.01$). Pre refers to the iron deficiency anemia (IDA) patient group, and Post refers to the group receiving iron supplementation and recovering from IDA. The superscripted a, b, and c denote the groups subjected to significance determination (a, Normal-Pre; b, Pre-Post; c, Normal-Post).
